# Hyperemia in head injury: can transcranial doppler help to personalize therapies for intracranial hypertension?

**DOI:** 10.3389/fneur.2023.1259180

**Published:** 2023-11-15

**Authors:** Camilla Gelormini, Eleonora Ioannoni, Angela Scavone, Luca Pisapia, Francesco Signorelli, Nicola Montano, Marco Piastra, Anselmo Caricato

**Affiliations:** ^1^Neurointensive Care Unit, Department of Anesthesiology, Intensive Care and Emergency Medicine, Fondazione Policlinico Universitario “Agostino Gemelli” IRCCS, Rome, Italy; ^2^Neurosurgery Section, Department of Neuroscience, Fondazione Policlinico Universitario “Agostino Gemelli” IRCCS, Rome, Italy; ^3^Neurosurgery Section, Department of Neuroscience, Università Cattolica del Sacro Cuore, Rome, Italy; ^4^Pediatric ICU and Trauma Center, Department of Anesthesiology, Intensive Care and Emergency Medicine, Fondazione Policlinico Universitario “Agostino Gemelli” IRCCS, Rome, Italy; ^5^Department of Anesthesiology, Intensive Care and Emergency Medicine, Università Cattolica del Sacro Cuore, Rome, Italy

**Keywords:** transcranial doppler, traumatic brain injury, cerebral hyperemia, intracranial hypertension, cerebral autoregulation

## Abstract

**Introduction:**

An increase in cerebral blood flow is frequent after traumatic brain injury (TBI) and can lead to brain swelling and refractory intracranial hypertension. We hypothesized that Transcranial EcoDoppler (TCD) monitoring could be useful to detect the cause of intracranial hypertension in these patients. Our main objective was to investigate if the increase of velocity in the middle cerebral artery (MCA) on TCD could be associated with intracranial hypertension.

**Methods:**

We retrospectively studied TBI patients consecutively monitored with TCD. Hyperemia was defined as MCA mean velocity higher than 80 cm/s. Intracranial hypertension was considered when hyperosmolar therapy, hyperventilation, or deep sedation was used.

**Results:**

We found hyperemia in 40 patients out of 118 (33.9%). On average, it started at day 2.1 ± 0.9 from admission and significantly increased (MCA velocity at day 1: 74 ± 25 cm/s vs. 109 ± 36 cm/s at day 4; *p* < 0.001). Intracranial hypertension was significantly associated with hyperemia, occurring in 92.5% of hyperemic and 51.3% of non-hyperemic patients (*p* < 0.001). Moreover, we found that hyperemia preceded severe intracranial hypertension (*p* < 0.0001). In a logistic regression model, hyperemia was the only variable significantly correlated with intracranial hypertension (OR 10.64; *p* < 0.001).

**Discussion:**

Hyperemia was frequent in our population of TBI patients and preceded intracranial hypertension. TCD monitoring, if performed on a daily regular basis, can be a useful method to detect this phenomenon and to guide the therapy. It could be a tool for a cause-oriented therapy of intracranial hypertension.

## Introduction

Management of severe head trauma is based on the prevention of secondary damage. In the first phase after trauma, it mainly consists of the early detection of hypoxia and hypotension; afterwards, one of the most important treatment is the control of intracranial pressure and the maintenance of adequate cerebral perfusion pressure. No accurate bedside measurement of cerebral blood flow is possible to date, and surrogate evaluation of flow through pressure is recommended. In this setting, we have no definitive tools to understand if cerebral perfusion pressure is adequate. Recently, several authors hypothesized that a normal response of arteriolar caliber to the variation of cerebral perfusion pressure, the so-called autoregulation, could be a marker of adequacy of flow. On the other side, when this response is altered, an increase in cerebral perfusion pressure can induce an increase in cerebral blood flow. This phenomenon is frequently observed after traumatic brain injury (TBI) and can lead to brain swelling and refractory intracranial hypertension (1–5).

Transcranial eco-Doppler (TCD) offers a non-invasive and easily reproducible method for evaluating cerebral hemodynamics (6, 7) and was suggested as a device to investigate the adequacy of bedside assessment of cerebral blood flow. We hypothesized that it could be a useful method to detect early derangements of cerebral blood flow (CBF) after trauma. Our main objective was to study if the increase of velocity in the middle cerebral artery (MCA) on TCD could be associated with intracranial hypertension. The secondary objective was to describe the clinical variables associated with the presence of an increase in MCA velocity.

## Materials and methods

### Study design and patient selection

In this monocentric retrospective study, we included traumatic brain injured patients consecutively admitted from May 2017 to January 2023 in the Neurointensive Care Unit of “A. Gemelli” University Hospital in Rome. The inclusion criteria were as follows: age > 18 years, GCS on admission in Emergency Department ≤8, at least one TCD recording every 24 h during the first 96 h after admission in the Neurointensive Care, Intracranial pressure (ICP) monitoring. Excluding criteria were as follows: Lindegaard index >3 or CT angiography suggestive of vasospasm, patients who died in the first 48 h. By using a threshold of 80 cm/s as TCD mean velocity in MCA, patients were divided into two groups: hyperemic and not hyperemic. A condition of intracranial hypertension was considered present if hyperosmolar therapy (first tier), hypocapnia (second tier), or deep sedation (third tier) was administered, according to ICP results. Consent for participation was waived due to the retrospective design of the study, and since no patient information was extracted except for research purposes. Anonymity was guaranteed.

### Data source

All eligible patients’ electronic medical records were reviewed by two researchers (CG, LP). Age, type of intracranial injury, bilateral MCA values on TCD, natremia, ICP, Glasgow Coma Scale (GCS) score at the admission in neuro-ICU and daily thereafter, therapies for ICP control and neurosurgical treatment were recorded for subsequent analysis. Confounders such as hyperthermia, hypovolemia, hematocrit and systemic abnormalities, hypercapnia, hypocapnia not driven by ICP control, glycemia disorders were excluded before including the data collected. Based on intracranial injury, different types of lesions were observed (Table 1).

**Table 1:** Descriptive statistics.

Hyperemic						Not hyperemic					
	Mean	SD	Min.	Max.	N. Tot. Obs	Mean	SD	Min.	Max.	N. Tot. Obs	*p*-value
Age (years)	42.0	20.7	18	84	40	56.2	21.0	19	90	78	0.0007**
GCS	6.1	3.0	3	14	40	7.6	3.8	3	15	78	0.03*
Initial MCA velocity (cm/s)	74.5	25.6	26.3	150.6	40	49.6	15.6	27.2	79.1	78	0.0001**
Higher MCA velocity (cm/s)	127.4	41.2	80.8	257.3	40	62.2	14.4	27.7	79.5	78	0.0001**

Min, minimum value; Max, maximum value; N. Tot. obs, total number of observations.

**p < 0.01; *p < 0.05.

We assumed as hyperemic all those patients who presented an MCA velocity above 80 cm/s in the period of observation, after excluding extracranial causes (8–10). Only the higher velocity was considered for analysis when abnormal MCA velocities were recorded on both sides.

### Multimodal neuromonitoring

All TCDs were performed in NeuroIntensive Care after admission by an experienced sonographer (CG, EI). Toshiba Xario 200 with a 2 MHz probe and a dedicated preset was used. The temporal and submandibular sonographic window was used to record velocities in MCA and extracranial internal carotid arteries (ICA) respectively. ICP monitoring was part of neurological multimodal monitoring and included intraparenchymal, subdural, or ventricular systems.

### Statistical analysis

Data were analyzed using Stata software V.14.1 by two authors (CG, AC). Anova for multiple comparisons was carried out for quantitative variables, *t*-test and chi-square test were carried out for comparisons between groups for categorical variables, as appropriate. Results were expressed as means ± standard deviations (SD). To analyze the utility of several variables for predicting the development of intracranial hypertension, an order logistic regression model was performed. For all tests, statistical significance was considered as *p* < 0.05.

## Results

We found 376 severe head injured patients in our database. Of these, 118 were considered for the analysis. Missing TCD values was the most frequent cause for exclusion. This occurred in 144 cases, due to the unavailability of the TCD system. Other conditions included death before 48 h (11 patients), presence of vasospasm (2 patients), absence of intracranial pressure monitoring (45 patients), and other confounders (such as hyperthermia, hypovolemia, or carbon dioxide disorders, 56 patients). In the study group, 40 out of 118 (33.9%) were classified as hyperemic, while 78 patients as not hyperemic. The most common lesions were focal contusions (20%) and acute subdural hematoma (47%), with no difference between groups (χ^2^ = 2.113; *p* = 0.715).

**Figure 1:**
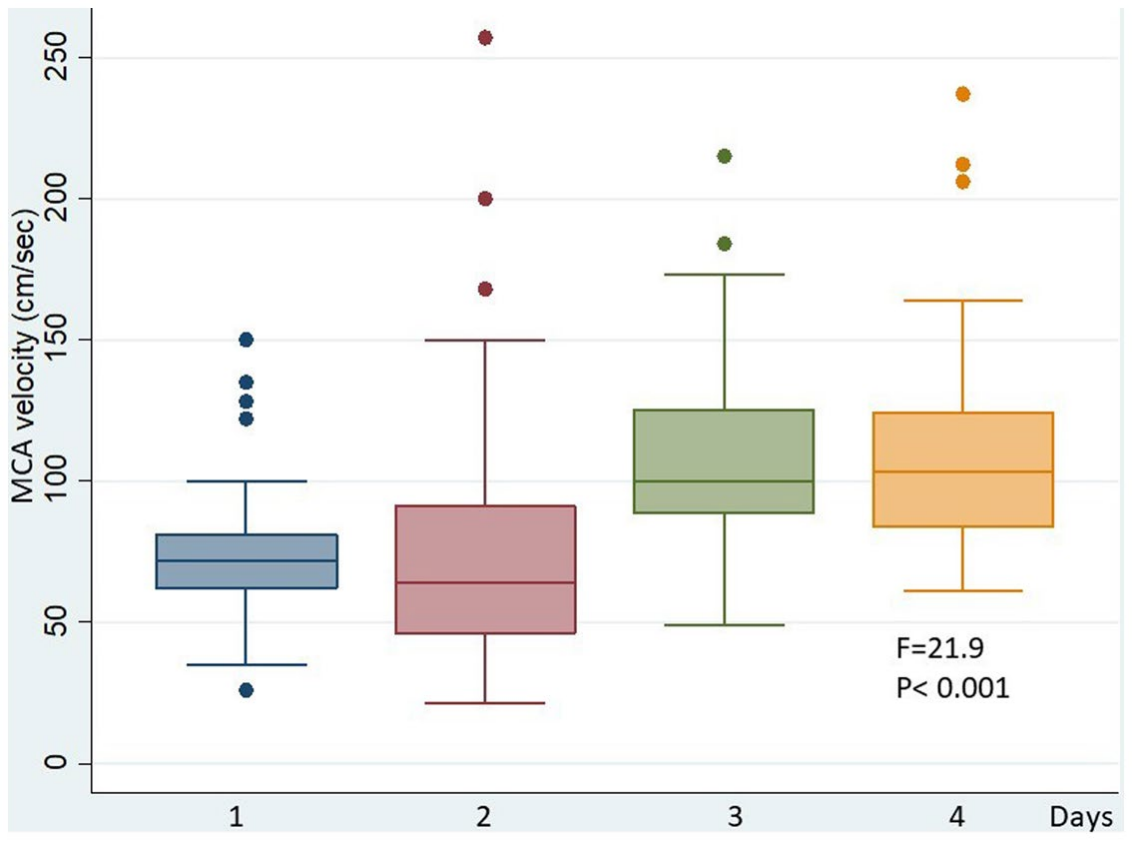
MCA velocity in the hyperemic group during the days. In these patients, TCD velocities significantly increased during the ICU staying.

On average, hyperemia started at day 2.1 ± 0.9 from admission, and increased during the stay in ICU for the next days (MCA velocity was 74 ± 25 cm/s at day 1 vs. 109 ± 36 cm/s at day 4; *F* = 21.9; *p* < 0.001) (Figure 1). This was more frequent in younger patients (42.0 ± 20.7 vs. 56.2 ± 21.0; *t* = 3.4715; *p* < 0.001) and in the most severe lesions (GCS on admission 6.1 ± 3.0 vs. 7.6 ± 3.8; *t* = 2.172; *p* < 0.05) (Table 1). Hyperemia was associated with high-level treatment for intracranial hypertension, requiring third-level therapy more frequently than in not hyperemic patients. This occurred in 37 of 40 patients (92.5%) in the hyperemic group, and in 40 of 78 patients (51.3%) in the non hyperemic group; (χ^2^ = 19.812; *p* < 0.001). Only 3 hyperemic patients did not need therapies for high ICP.

**Figure 2:**
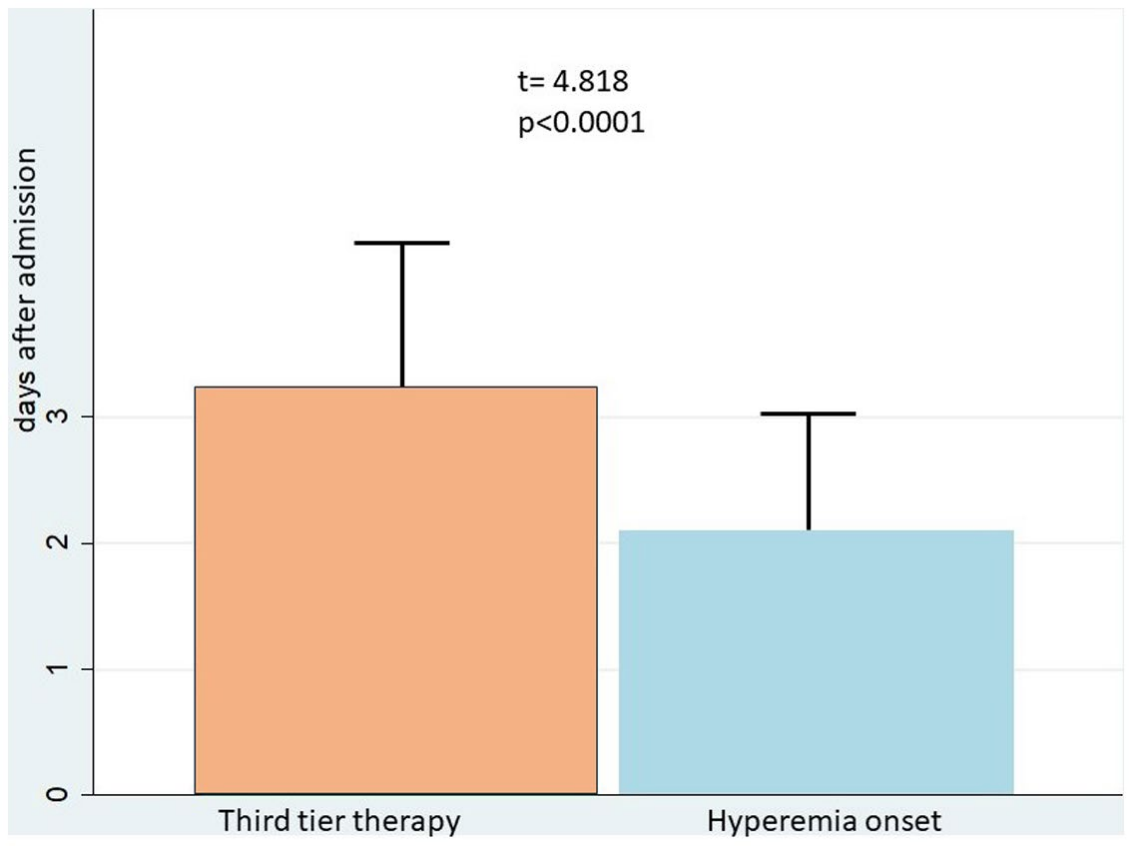
Hyperemia significantly preceded the development of severe intracranial hypertension.

Moreover, we found that hyperemia occurred on average 1 day prior to severe intracranial hypertension: third tier therapy for ICP control started at day 3.2 ± 1.1, while the diagnosis of hyperemia was earlier (day 2.1 ± 0.9; *t* = 4.818, *p* < 0.0001) (Figure 2).

By using a model of logistic regression, hyperemia was the only parameter significantly correlated with intracranial hypertension (OR 10.64; *p* < 0.001) (Table 2).

**Table 2:** Model of logistic regression.

Intracranial hypertension	Odds ratio	Std. Err.	*z*	*p*	95% conf. interval
**Hyperemia**					
No	1	(Base)			
Yes	10.64	7.62	3.30	0.001	2.62 – 43.29
Higher V MCA	0.99	0.006	−1.02	0.310	0.98 – 1.00
**Pathologies**					
Contusions	1	(Base)			
Subdural	2.38	1.29	1.59	0.112	0.82 – 6.89
Epidural	2.23	1.83	0.98	0.329	0.45 – 11.15
Subarachnoid	0.50	0.38	−0.91	0.362	0.11 – 2.24
DAI	0.41	0.45	−0.81	0.420	0.05 – 3.61
GCS on admission	0.93	0.06	−1.15	0.251	0.82 – 1.05

*N* = 118.

LR χ^2^= 33.19.

*p* < 0.00001.

Pseudo *R*^2^ = 0.2177.

Log likelihood = −59.616491.

*z* = ratio of the estimated coefficient to its standard error.

Therapy for intracranial hypertension was considered as dependent variable.

## Discussion

The main result of this study was that hyperemia was frequently observed by TCD in patients with a severe head injury. In these cases, MCA velocities increased in the days following the diagnosis and were associated with intracranial hypertension. Hyperemia on TCD significantly preceded intracranial hypertension and was associated with the severity of trauma and younger age.

Measurement of cerebral blood flow after trauma has been the object of several studies in recent years and conflicting results have been reported (11–13). Since more sophisticated imaging techniques have become available, including Positron Emission Tomography (PET), the pathophysiology of brain hemodynamics is now better understood (4). In particular, several studies have shown that brain ischemia was a common finding in the first 24 h after a head injury, and the outcome could improve when a bundle aimed to avoid further reduction of cerebral blood flow was implemented (2–4, 14). After this phase, hyperemia was observed 1–3 days after trauma, and it was associated with generalized abnormalities in flow-metabolism coupling. Seminal studies from the Cambridge group by PET observed that brain regions with low oxygen extraction suggesting hyperemia were common and prominent between days 2 through 5, and this pattern was associated with an increase in cerebral blood volume. They showed that these vascular abnormalities were one of the most important determinants of intracranial hypertension, providing a physiological basis for intervention aimed at reducing the vascular contribution to intracranial volume (4).

Previous papers on TCD should be regarded according to these important results (2, 3, 11, 13, 15). In particular, an increase of cerebral blood flow velocity was observed by TCD in the days 1–3 after trauma. Martin et al. studied 125 head injured patients with TCD and CBF measurements (2). They showed that low velocities on TCD were frequent in the first 24 h after trauma and an increase of TCD velocities in the hyperemic range (86 ± 4 cm/s) occurred in days 1–3. Unfortunately, correlation with ICP or prognosis was not investigated. Other authors have studied this topic in small case series. Zurynski et al. observed that in 50 patients with head trauma, hyperemia on TCD was associated with intracranial hypertension, low cerebral perfusion pressure, and poor outcome (16). The same results were found by Muttaqin et al. on 35 head-injured patients (15).

By using PET at days 1–3 after trauma, Launey et al. found high values of CBF in the presence of a reduced metabolic rate of oxygen, confirming the presence of true hyperemia (4). This is an important point since TCD is a non-invasive method, the result of which could be affected by several confounding factors. In particular, the increase of velocities could be the effect of spasm, rather than hyperemia; moreover, the reduction of caliber of small vessels can occur as a result of many events, such as intracranial hypertension, distal vasospasm or arterial hypocapnia, and can affect TCD results. Many of these events could be induced by trauma as a primary consequence, and others could be the effect of the treatment. In this setting, only sequential examinations of sophisticated imaging studies based on metabolic techniques can help to understand basic physiology and guide the treatment.

On the other hand, these methods are not available everywhere, and we need practical bedside tools to understand these phenomena. In this study, we used TCD daily after trauma and considered hyperemia when the increase of cerebral blood flow velocity was associated with Lindegaard index <3 or with the absence of large arterial vasospasm on angio CT (17–20). Our results suggested that hyperemia was present in about one third of patients and that in these cases intracranial hypertension was very common at that time or in the following days, according to a further increase of cerebral blood flow velocities. Frequent examinations should be mandatory by TCD, to reduce mistakes in reading these data. This could be the case, in particular when intracranial hypertension occurs together with hyperemia. In these situations, the sum of these effects could give very different patterns of velocities on TCD and could explain conflicting interpretations of data in previous reports.

Taken together with PET studies, our data suggest that an abnormal flow-metabolism coupling could be present, and an autoregulation test should be performed to guide the treatment. This could have important clinical implications, since therapies aimed at controlling hyperemia, such as the strict control of CPP in the lower range of autoregulation, cautious use of mild hypocapnia, and an analgosedation targeted on monitor tools that avoids sudden increase of metabolism, could decrease the extent of intracranial hypertension.

Several limitations should be considered in this study. First of all, this was a retrospective analysis, and many missing data, mainly due to the unavailability of the TCD, reduce the power of these results. Furthermore, the diagnosis of hyperemia was based on TCD observations and the calculation of the Lindegaard index, confirmed by angio-CT in not all the cases. The threshold value was considered, based on previous observations (10). The autoregulation test was not part of a standardized protocol, and data were not sufficient to be analyzed.

## Conclusion

Hyperemia was frequent in our population of patients with severe traumatic brain injury and significantly preceded intracranial hypertension. TCD monitoring, if performed on a daily regular basis, can be a useful method to detect this phenomenon and to guide the therapy. It could be a tool for a cause-oriented therapy of intracranial hypertension.

## Data availability statement

The raw data supporting the conclusions of this article will be made available by the authors, without undue reservation.

## Ethics statement

The studies were conducted in accordance with the local legislation and institutional requirements. Written informed consent for participation was not required from the participants or the participants’ legal guardians/next of kin because consent for participation was waived for the retrospective design of the study, and since no patient information was extracted except for research purpose. Anonymity was guaranteed.

## Author contributions

CG: Data curation, Formal analysis, Methodology, Software, Writing – original draft. EI: Data curation, Writing – review & editing. AS: Data curation, Methodology, Writing – review & editing. LP: Data curation, Writing – original draft. FS: Supervision, Writing – review & editing. NM: Supervision, Writing – review & editing. MP: Supervision, Writing – review & editing. AC: Conceptualization, Data curation, Methodology, Supervision, Writing – original draft, Writing – review & editing.
